# Thiamine deficiency in Gambian women of reproductive age

**DOI:** 10.1111/nyas.14695

**Published:** 2021-09-20

**Authors:** Megan W. Bourassa, Filomena Gomes, Kerry S. Jones, Albert Koulman, Andrew M. Prentice, Carla Cerami

**Affiliations:** ^1^ Nutrition Science The New York Academy of Sciences New York New York; ^2^ NIHR BRC Nutritional Biomarker Laboratory, MRC Epidemiology Unit University of Cambridge Cambridge United Kingdom; ^3^ MRC Unit The Gambia at London School of Hygiene & Tropical Medicine Banjul The Gambia; ^4^ NOVA Medical School Lisbon Portugal

**Keywords:** thiamine, erythrocyte transketolase, thiamine deficiency disorders, beriberi, The Gambia, women

## Abstract

Thiamine deficiency disorders are associated with a variety of clinical symptoms affecting the nervous and cardiovascular systems. There is growing recognition that thiamine deficiency can occur in populations well beyond the classical region of South Asia, and at‐risk populations include those who receive a large proportion of their energy from polished white rice (or other low‐thiamine staple foods) and with low dietary diversity. Reports of thiamine deficiency in West Africa over the last century have suggested that this has historically been an issue in this population, but in more recent decades, these reports have been limited to prison populations. To understand if thiamine deficiency might be an unrecognized problem in the communities of this region, erythrocyte samples collected during the wet and dry seasons from 226 women of reproductive age (mean age = 28 years old) were assessed for thiamine status by measuring the erythrocyte transketolase activity coefficient (ETKac). Overall, 35.8% of the sample was at high risk of thiamine deficiency (ETKac ≥ 1.25). Risk of thiamine deficiency was significantly higher in the wet (47.9%) compared with the dry season (22.9%) (*P* < 0.001). To our knowledge, this is the first report of biochemical thiamine deficiency in a free‐living population in West Africa in the 21st century and suggests that further investigation is warranted.

## Graphical abstract

To understand if thiamine deficiency might be an unrecognized problem in West Africa, erythrocyte samples collected during the wet and dry seasons from Gambian women of reproductive age were assessed for thiamine status by measuring the erythrocyte transketolase activity coefficient. To our knowledge, this is the first study of biochemical thiamine deficiency in a free‐living population in West Africa in the 21st century.

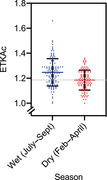

## Introduction

In recent decades, reports of thiamine deficiency in sub‐Saharan Africa have largely been in isolated cases, including groups of refugees,^1^ prisoners,^2^, ^3^ soldiers,^4^ and those in remote communities.^5^, ^6^ As a result, thiamine deficiency, which is most commonly described in Southeast Asia, is generally not considered a problem in Africa. However, there is recent evidence that thiamine deficiency is much more widespread than previously believed, and its vague and variable clinical presentations make it easy to overlook or misdiagnose.^7^ For example, thiamine deficiency was recently identified as the cause of a frequent and nearly always fatal illness in infants and postpartum women in Northern India, where it went undetected and undiagnosed for decades.^8^ This case, as well as others, are well outside the classical geographic locations associated with thiamine deficiency and have, therefore, raised questions about the true global prevalence of thiamine deficiency.^9^, ^10^, ^11^, ^12^ In areas where thiamine deficiency is a known problem, it can have a serious impact on infant mortality. For example, in Myanmar, deficiency of this essential vitamin is the second most common cause of death in infants.^13^ While exclusively breastfed infants (from thiamine‐deficient mothers) are at the greatest risk of mortality from thiamine deficiency, it can also be fatal in adults if not efficiently diagnosed and treated.^12^, ^14^

Thiamine deficiency is most prevalent among communities with a high carbohydrate diet, primarily consisting of polished white rice, along with low intake of thiamine‐containing foods.^14^ It can also be exacerbated in conditions with heavy physical labor,^15^ which increase thiamine requirements, or through the consumption of antithiamine‐containing foods, such as raw or fermented fish.^15^ Over the last several decades, rice consumption has increased dramatically in parts of Africa, and in some areas, rice is consumed at levels similar to what is seen in Southeast Asia.^16^ The more traditional grains consumed in West Africa, including millet and sorghum, contain thiamine but experience a 20–30% decrease in thiamine content after cooking because of the properties of this vitamin, that is, water solubility and susceptibility to heat.^16^, ^17^ Seasonal variability in diets can also limit dietary diversity, especially in rural areas in the wet season when fresh foods can be more scarce and physical labor, associated with subsistence farming, increases.^18^, ^19^, ^20^ These factors contribute to a greater risk of thiamine deficiency and therefore, there is a need to examine thiamine status in sub‐Saharan Africa.^20^, ^21^

The Gambia, like much of West Africa, experiences a rainy season between June and November and a dry season from December through May.^22^ During the dry (harvest) season, more fresh foods are available (from postharvest income and borehole‐irrigated market gardening), and there is more time in the day for women to cook.^23^ By contrast, in the rainy season, women spend more time involved in intense physical labor, and diets become both more restrictive and less abundant (because food stocks run low before the next harvest).^22^ In 1988, an outbreak of thiamine deficiency in a Gambian village was attributed to an excessively rainy wet season leading to an increase in farm work load and a decrease in income from work outside the village.^24^ Subsequent studies showed a strong seasonal variability in thiamine intake.^24^ During the dry season, the availability of thiamine‐containing foods, such as groundnuts and cassava leaves, is more plentiful than during the rainy season.^22^ Therefore, in the current study, we aimed to assess the thiamine status of women of reproductive age (WRA) living in rural Gambia and determine the association between thiamine status and seasonality.

## Materials and methods

Washed erythrocyte samples from 226 WRA (18–40 years old) were obtained from the Keneba Biobank at the MRC Unit in The Gambia of the London School of Hygiene and Tropical Medicine. Blood samples had been collected between 2014 and 2016 during the middle to latter half of either the rainy season (July through September) or the dry season (February through April) and were collected for the biobank before the design of this study. All inhabitants of the West Kiang region were invited to donate blood to the biobank and over 95% agreed. Participants were not asked at the time of sample collection if they were pregnant or lactating. However, on the basis of delivery dates in the medical database before and after the sample collection, we can estimate that 80 women were lactating and 33 were pregnant. This estimate assumes a duration of pregnancy of 280 days and average local lactation period of 21 months. Owing to the uncertainty of this information, thiamine status of these subgroups was not further analyzed. None of the samples were collected during Ramadan. Participants had fasted overnight and venous blood samples were collected into EDTA‐containing blood tubes. The sample was centrifuged, the plasma was removed, and erythrocytes washed three times with an equal volume of normal saline (0.9% w/v NaCl). Washed erythrocytes were stored at −70 °C.

Samples were shipped from The Gambia on dry ice and analyzed for thiamine status on the basis of the erythrocyte transketolase activity coefficient (ETKac) at the NIHR Nutritional Biomarker Laboratory at the MRC Epidemiology Unit, University of Cambridge, using the protocol by Jones *et al*.^25^ ETKac is a ratio of activated to basal activity of the thiamine‐dependent transketolase enzyme. Thus, a higher (unitless) activity coefficient indicates more severe thiamine deficiency; values greater than 1.25 indicate high risk of thiamine deficiency, values between 1.15 and 1.25 indicate moderate risk, and values below 1.15 suggest low risk. Although there is no consensus on cutoff values for ETKac, these values and cutoffs are the most commonly used, and clinical symptoms of thiamine deficiency are generally associated with even higher values (∼1.4).^14^, ^26^

Mann–Whitney and Kruskal–Wallis tests were used to assess the association between thiamine status (continuous variable) and anemia status, age, and body mass index (BMI). The Pearson product‐moment correlation coefficient was also computed to assess the relationship between these variables. The chi‐square test was used to compare the differences in categorical variables (e.g., rate of women at risk of thiamine deficiency) between the wet and dry seasons. Statistical analyses were carried out using SPSS software version 27 for Windows (Chicago, IL). Any differences were considered significant when the *P* value was less than 0.05.

### Ethics approval

Collection of samples for the West Kiang Biobank (SCC/EC1185, SCC/EC2012.3, and SCC/EC2013.53) and subsequent approval for this analysis (SCC/EC 17574) were approved by the MRCG Scientific Coordinating Committee (SCC) and the joint Gambian Government/MRCG Ethics Committee. All subjects signed an informed consent form after full explanation of the biobanking procedures and subsequent sample/data usage.

### Data sharing

The data will be openly available. Requests should be submitted to the corresponding author.

## Results

Participant characteristics are shown in Table 1. Nearly, all were of the Mandinka ethnicity (99%), which is the majority group (∼79.9%) of West Kiang.^27^ They are also largest ethnic group in The Gambia, constituting 34.4% of the population.^28^ The mean age was 28 years old (range: 18–40 years old). Most women (70.4%) had a normal weight (based on BMI of 18.5–25 kg/m^2^), and 59.7% of the women were anemic (hemoglobin <12 g/dL). Just over half of the samples (51.8%) were collected between July and September, during the wet season, while the remainder of the samples (48.2%) were collected between February through April.

**Table 1:** Sample population characteristics

Variable	*n* = 226
Age, years	
Mean (SD)	28 (6.9)
Range	18−40
Body mass index (BMI), kg/m^2^	
Mean (SD)	28.6 (4.0)
Range	14.8−43.4
Underweight (BMI < 18.5), (*n*, %)	35, 15.5%
Normal weight (BMI: 18.5−25), (*n*, %)	159, 70.4%
Overweight (BMI: 25−30), (*n*, %)	23, 10.2%
Obese (BMI > 30), (*n*, %)	9, 4.0%
Hemoglobin, g/dL	
Mean (SD)	11.6 (1.4)
Anemic, <12 g/dL (*n*, %)	135, 59.7%
Season of sample collection	
Wet (*n*, %)	117, 51.8%
Dry (*n*, %)	109, 48.2%
Year of sample collection	
2014 (*n*, %)	29 (12.8%)
2015 (*n*, %)	117 (51.8%)
2016 (*n*, %)	80 (35.4%)
Ethnicity	
Mandinka (*n*, %)	224 (99%)
Wollof (*n*, %)	1 (0.5%)
Other (*n*, %)	1 (0.5%)

**Figure 1:**
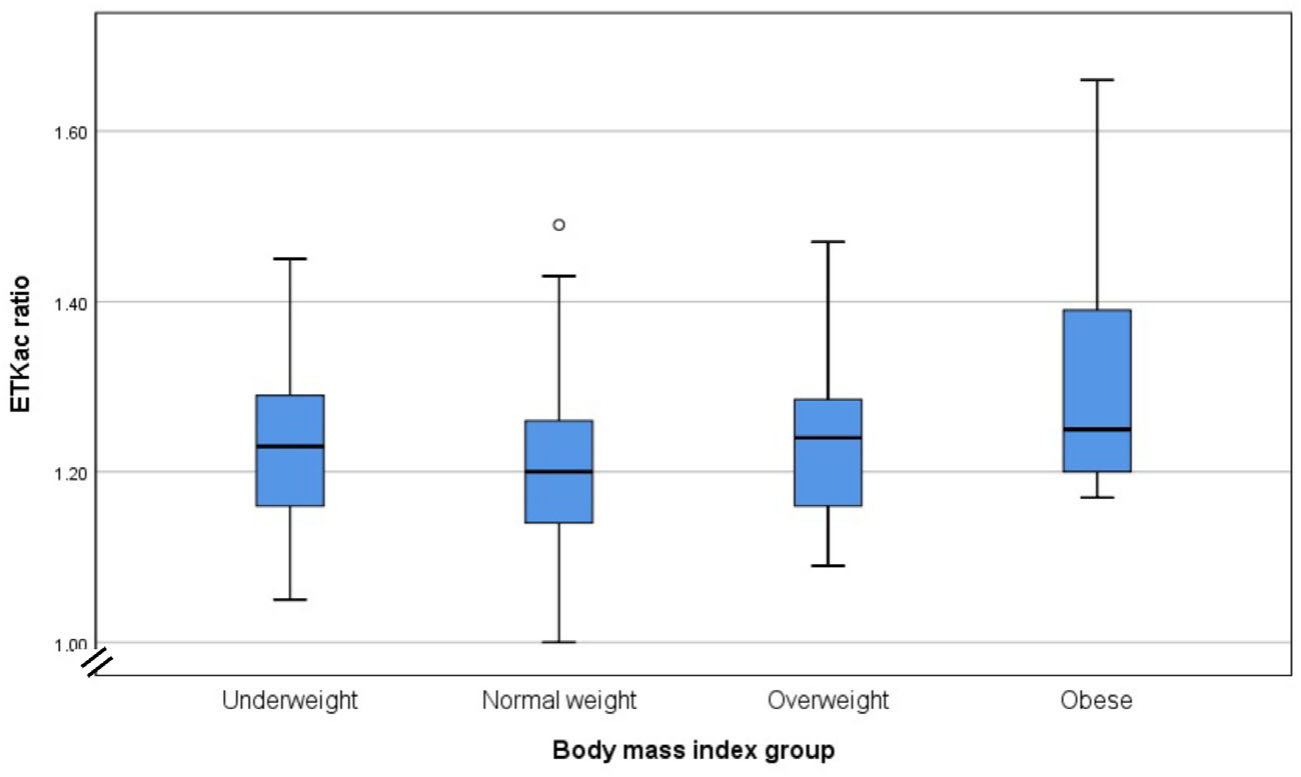
Thiamine status (median ETKac values) of WRA according to groups of body mass index.

Thiamine status was assessed with the ETKac. On the basis of the Shapiro–Wilk test, the ETKac data were not normally distributed (*P* = 0.001), and, as such, nonparametric tests were used. The median ETKac of all the samples (*n* = 226) was 1.21 (range: 1.00–1.66), suggesting a moderate risk of deficiency overall in WRA. Among these samples, 35.8% had a value greater than 1.25, indicating a high risk of deficiency. There were no statistically significant differences in the median ETKac levels between anemic and nonanemic women (Mann–Whitney test, *P* value = 0.22), or between younger (<28 years old) and older women (≥28 years old) (Mann–Whitney test, *P* value = 0.48), but there was a trend for a significant difference between the four BMI categories (Kruskal–Wallis test, *P* value = 0.07). Obese women (*n* = 9) were at higher risk of thiamine deficiency (median ETKac = 1.25, range: 1.17–1.66), followed by overweight women (median ETKac = 1.25, range: 1.17–1.66) and underweight women (median ETKac = 1.23, range: 1.05–1.45), while normal weight women presented lower levels of ETKac (median ETKac = 1.20, range: 1–1.49) (Fig. 1).

**Figure 2:**
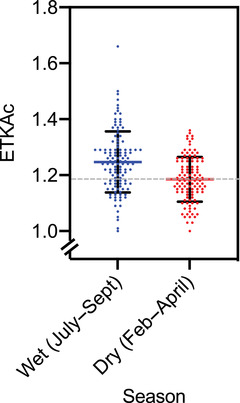
Thiamine status of Gambian women of reproductive age in the wet and dry seasons, which was statistically significant (Mann–Whitney test, *P* value < 0.001). The dashed gray line is at an erythrocyte transketolase activity coefficient (ETKac) value of 1.25, and values at or above this line are at high risk of thiamine deficiency.

When samples were divided by the season of collection, there was a significantly greater risk of thiamine deficiency during the wet season when compared with the dry season (chi‐square test, *P* value < 0.001). In the wet season, the median (range) ETKac was 1.24 (1–1.66), with 47.9% of participants above the cutoff of 1.25, indicating high risk of deficiency (Fig. 2). In the dry season, the median value was significantly lower, indicating better thiamine status at 1.18 (1–1.36), and only 22.9% of participants had ETKac values associated with a high risk of deficiency (chi‐square test, *P* value < 0.001). The differences in the median ETKac values between the dry and wet seasons were statistically significant (Mann–Whitney test, *P* value < 0.001).

There were no significant differences in ETKac between wet season samples collected in different years between 2014 and 2016, nor in the dry season samples collected in 2015 and 2016.

## Discussion

Using ETKac to assess thiamine status, we found in this study population of WRA living in rural Gambia that more than one third (35.8%) were at high risk (ETKac ≥ 1.25) of thiamine deficiency. While comparative data from other countries are fairly limited, the current levels of deficiency are worse than in areas where thiamine deficiency is a more commonly recognized problem, such as Southeast Asia. For instance, as part of the national micronutrient survey in Cambodia, it was found that 27% of WRA were thiamine deficient (using a conservative ThDP cutoff of <120 nmol/L).^29^ In a recent trial also in Cambodia, 25% of women in the placebo group who did not receive thiamine supplements were at high risk (ETKac ≥ 1.25) of deficiency at 24 weeks postpartum.^30^ In a population of Karen refugees in Thailand, it was observed that 26.9% (7/26) of women 3 months postpartum were at high risk (ETKac ≥ 1.25) of thiamine deficiency.^31^ Each of these examples are in postpartum and mostly lactating women, who have higher thiamine requirements than nonpregnant, nonlactating women. However, our data suggest that the thiamine status of women in this sample population is worse than in areas where thiamine deficiency is known to occur or where an outbreak has occurred. The wet season appears to further increase this risk to nearly half the sample population.

In the 20th century, there were several reports of thiamine deficiency in The Gambia, but the most recent of these reports are from the late 1980s and early 1990s in free‐living adults.^32^, ^33^, ^34^, ^35^ In 1988, at least 140 people presented to a rural clinic in the North Bank Division of The Gambia in the early portion of the rainy season with symptoms of thiamine deficiency.^24^ A few years later, from 1990 to 1991, 38 patients were seen in an urban hospital setting in the capital, Banjul, with a peak during the latter half of the rainy season.^35^ The authors concluded that low dietary intake of thiamine and specifically the consumption of imported and unfortified polished white rice were contributing factors for thiamine deficiency, especially during the rainy season when fresh foods become scarcer and more expensive. Despite these reports, the literature on thiamine deficiency in this area is very limited, and there is no information available on the clinical presentation of symptoms. However, the data presented here suggest that thiamine deficiency may continue to be a problem in The Gambia 30 years after these reports.

The samples for the present analysis come from a well‐studied population in West Kiang.^27^ For several decades, numerous dietary studies have been undertaken to understand the seasonal variability of diets on micronutrient intakes and status.^21^, ^22^, ^24^ However, thiamine has not been studied in West Kiang and is notably absent from the Gambian food composition table.^36^ In this population, the staple grain is typically rice, which is consumed with very small amounts of meat, usually beef, goat, poultry, or fish and a sauce made of groundnuts (peanuts) or leaves and/or tomatoes.^37^ Mangos, oranges, cassava, and groundnuts are commonly consumed as snacks when they are seasonally available. While mangos and oranges are only available during a limited season, cassava and groundnuts may be available in limited quantity as what remains from the previous year's harvest is consumed. On the basis of the available information, the largest sources of thiamine consumed by the population come from groundnuts, beans, and oranges, which are available during the dry season but become scarce during the wet season. There is no apparent consumption in this region of antithiamine compounds, including, betel nut, raw or fermented fish, and silkworm larvae.

Table 2 lists some of the most commonly consumed foods in West Kiang, with their thiamine content per 100 g, based on the West African Food Composition Database and seasonal availability.^17^ It is clear that the staple grains do not contain much thiamine, especially after cooking. The richest sources of thiamine are the legumes, which are consumed primarily in the dry season, as are many of the fruits and vegetables, which also contain thiamine. The meats, which are consumed in small quantities, contain relatively little thiamine. Pork contains relatively high amounts of thiamine (0.61 mg/100 g of edible food), but in this primarily Muslim population, pork is not consumed. While this is not intended to be a comprehensive list of thiamine‐containing foods consumed in The Gambia, it suggests that there is limited availability for the diet to meet the 1.1 mg/day recommended dietary allowance (RDA) for WRA, especially in the wet season. Importantly, this RDA is based on the U.S. population and does not account for the increased needs of pregnancy or lactation, during which thiamine requirements increase to 1.4 mg.^38^ It is also estimated that thiamine requirements are higher in individuals who undertake heavy manual labor, such as the farming that rural Gambian women participate in during the wet season.^39^

**Table 2:** Thiamine content and seasonal availability of commonly consumed foods in The Gambia

	Thiamine content mg/100 g of edible food	Seasonal availability
Cereals		
Rice, white, polished, boiled	0.01	Wet and dry
Millet, decorticated, boiled	0.04	Wet and dry
Maize, white, whole kernel, boiled	0.05	Wet and dry
Fruit		
Mangoes, raw	0.04	Dry
Oranges, raw	0.03	Dry
Tomatoes, raw	0.06	Dry
Legumes		
Locust beans, soaked, boiled	0.16	Dry
Groundnuts, shelled, dried, raw	0.87	Dry
Groundnut paste, from groundnuts only	0.39	Dry
Vegetables		
Pumpkin, light orange flesh, boiled, drained	0.06	Dry
Okra, boiled, drained	0.02	Dry
Aubergine/eggplant, boiled, drained	0.04	Dry
Cassava, white flesh, boiled	0.03	Wet and dry
Leafy greens		
Red sorrel (kucha), fresh, boiled, drained	0.21	Wet and dry
Cassava leaves (nyambi jambo), fresh, boiled, drained	0.24	Wet and dry
Baobab leaves (naa), fresh, boiled, drained	0.06	Wet and dry
Moringa (nebedayo)	0.24	Wet and dry
Meat		
Beef, lean, 5% fat, grilled	0.13	Wet and dry
Goat, lean, grilled	0.18	Wet and dry
Chicken, light meat with skin, grilled	0.07	Wet and dry
Fish		
Small tilapia, fillet, grilled	0.04	Wet and dry
Catfish, fillet, grilled	0.42	Wet and dry

This study also shows a trend for an increased risk of thiamine deficiency in obese women. This is not a surprising finding; despite being counterintuitive, obese individuals are considered a population at risk of thiamine deficiency. Obese individuals tend to have low intake of foods rich in thiamine (such as whole grains, pulses, nuts, and seeds), and they need higher amounts of thiamine to metabolize the typical dietary pattern heavy in carbohydrates, and in particular, simple sugars. This thiamine–energy/carbohydrate mismatch has been pointed out as the main contributing factor for thiamine deficiency in obese populations mostly in high‐income countries.^40^, ^41^, ^42^ However, the results from our study need to be interpreted with caution because the obese group was limited to only nine women.

Despite the poor biochemical thiamine status and likely low dietary intake of thiamine, it is possible that thiamine deficiency can go undetected in a population, which has recently been reported elsewhere in West Africa.^43^ Thiamine deficiency disorders have a wide range of manifestations that are easily confused with other ailments, from Wernicke encephalopathy, to edema, muscle weakness, and peripheral neuropathy.^44^ It has even been suggested that the seasonal ataxias seen in West Africa may be a result of thiamine deficiency.^45^ These neurological conditions peak during the wet season when diets become more limited and thiamine antagonists, such as African silk worm larvae, are consumed.^46^ There have been other recent examples from Southeast Asia, where hospitals have unknowingly seen patients with thiamine deficiency. In one such example in Assam, it was decades before the mysterious and often fatal illnesses observed in infants and postpartum women were accurately diagnosed and subsequently treated with thiamine.^8^ Another unexplained illness in Kiribati that primarily affected adult men was eventually identified to be caused by thiamine deficiency.^12^

The effects of thiamine deficiency in childhood may persist into later life. In Israel, a group of infants consumed infant formula in which the thiamine was inadvertently omitted.^47^ In the long‐term follow up of these children, serious motor and cognitive effects were recognized.^48^, ^49^ Subclinical or asymptomatic thiamine deficiency may also be cause for concern, especially for cognitive development of children. Infants who showed no clinical neurological signs of thiamine deficiency but had consumed the thiamine‐deficient formula have shown persistent deficits in language development at 5–6 years of age.^50^ Another cognitive study from a supplementation trial in Cambodia also showed that thiamine plays an important role in neurological development. In this study, the thiamine breastmilk content at 2 weeks of age was highly predictive of the infant's cognitive development up to 1 year of age, when the study concluded.^51^ While none of these exclusively breastfed infants showed clinical symptoms of thiamine deficiency, the breastmilk status was generally very low, as was the mother's blood thiamine status, and the study adds to the growing evidence that thiamine plays an important role in cognitive development. It also highlights the need to correct thiamine deficiency early, perhaps even preconception in WRA because of the sequestration of thiamine to the fetus, leading to higher thiamine requirements during pregnancy, and the role that thiamine plays in fetal development.^39^, ^52^

The main limitation of this study is that it is based on a convenience sample of 226 women from a large biobank of a population in Keneba and surrounding villages in the West Kiang region of The Gambia. It is unclear if this sample is representative of other areas in West Africa, The Gambia, or even other rural areas within the country. There is also some debate around the utility of biomarkers and their cutoffs for thiamine deficiency as they are not a good indicator of clinical symptoms.^53^, ^54^ The study also did not measure dietary thiamine intake. Lastly, there is no information on thiamine content in the Gambian Food Composition Table, and, as a result, estimates of foods contributing to dietary thiamine intake relied on the regional, West African Food Composition Table.^17^, ^36^

## Conclusions

While significantly more work needs to be done to understand the potential extent and impact that thiamine deficiency may have in rural Gambia, this is the first evidence of biochemical thiamine deficiency in free‐living people in West Africa in recent years. The observation that around half of women in the wet season are at high risk of thiamine deficiency is potentially significant and warrants further investigation. Diets in this population are likely very low in thiamine, and the reduced dietary diversity and high physical workload during the wet season appears to exacerbate the risk of deficiency. Thiamine deficiency can go unrecognized and undocumented because of the variability in clinical presentations. However, accurate diagnosis and prompt treatment is essential to limit the mortality and morbidity, including impacts on cognitive development that are associated with thiamine deficiency. The present work calls for additional population‐level studies on thiamine status and surveillance of thiamine deficiency disorders (at healthcare facilities) in The Gambia and other African countries that present similar risk factors for thiamine deficiency. A recent task force on thiamine deficiency has proposed that when more than 20% of the population has an ETKac above 1.2, then this should be considered a public health problem that warrants a large‐scale intervention.^14^ If further investigation shows that these data are representative of the larger population, it would suggest the need for an intervention.

## Author contributions

M.W.B. drafted the manuscript and designed the study with the help of C.C. and A.M.P. F.G. and M.W.B. analyzed the data. K.S.J. and A.K. oversaw the analysis of the ETKac and provided guidance with the dietary information in The Gambia. All authors contributed to the writing of the manuscript and have reviewed it.

### Peer review

The peer review history for this article is available at https://publons.com/publon/10.1111/nyas.14695

## Competing interests

The authors declare no competing interests.
